# Neglected intrauterine fetal demise for more than two decades leading to the development of a lithopedion: a case report

**DOI:** 10.1186/s13256-019-2264-8

**Published:** 2019-11-12

**Authors:** Fitsum Fikru Gebresenbet, Abdu Mengesha Mulat, Namus Muhajir Nur, Ferehiwot Bekele Getaneh

**Affiliations:** 1 Department of Radiology, Addis Ababa University, College of Health Sciences, Addis Ababa, Ethiopia; 2 Department of Obstetrics & Gynecology, Addis Ababa University, College of Health Sciences, Addis Ababa, Ethiopia

**Keywords:** Lithopedion, Fetal demise, Stone baby

## Abstract

**Background:**

Lithopedion is a word derived from the Greek words *lithos*, meaning stone, and *paidion*, meaning child, to describe a fetus that has become stony or petrified. Lithopedion is a rare complication of pregnancy which occurs when a fetus dies and becomes too large to be reabsorbed by the body. This entity in rare circumstances can be challenging for physicians to diagnose since it has a range of clinical manifestations.

**Case presentation:**

We present a case of a 55-year-old, gravida IV para III, Ethiopian woman from Ethiopia with a retained fetus and vesicovaginal fistula after an obstructed labor and a neglected intrauterine fetal demise of approximately 22 years. The diagnosis was confirmed by suggestive clinical history, physical examination findings, and an abdominopelvic computed tomography scan. Laparotomy and removal of the lithopedion was done and our patient was sent to a fistula hospital for vesicovaginal fistula repair.

**Conclusion:**

This case is a rare phenomenon in which the dead fetus remained in the uterus for a long time after a neglected obstructed labor and uterine rupture.

## Introduction

The word lithopedion is derived from Greek words to refer to a fetus that has calcified or changed to bone. This condition was first described in the tenth century by Albucasis, a surgeon of the Arabic era of medicine [1].

In the modern era of medicine, the occurrence of a lithopedion is extremely rare. Since the earliest case was discovered in 1582 in France, less than 300 cases of lithopedion have been reported. However, in places with limited access to health care facilities and poor health awareness, physicians may encounter this rare condition and its confusing clinical presentation [2]. Cases of lithopedion have been reported in women with an age range of 30 to 100 years and the estimated interval of lithopedion retention was in the range of 4 to 60 years [2].

Here we report a case of an obstructed labor 22 years ago which led to a uterine rupture, formation of a lithopedion, and a vesicovaginal fistula (VVF).

## Case presentation

Our patient is a 55-year-old Ethiopian woman, gravida IV para III abortion 0, who gave birth to three children at home by spontaneous vaginal delivery. She presented to our hospital claiming she was carrying a dead fetus in her womb from a pregnancy that had been there for 22 years. The pregnancy lasted up to nine months uneventfully at which time the membrane ruptured and she went into labor. After 3 days of labor at home, she developed vaginal bleeding and visited a nearby hospital where she was informed that she had a uterine rupture which had to be operated on. However, she refused the surgery and went home. Over time she developed urinary incontinence.

She lost all her three children from an unspecified medical illness before their first birthday, although she claimed all the pregnancies and the labor were uneventful. Since the incident, she is divorced and lives alone supported by her sister.

At her current presentation, she had lower abdominal pain which had increased in severity, vaginal discharge, and urine per vagina since the onset of the condition. She had no other known medical illness.

On physical examination there was a gravid uterus of 20-week size and a non-tender, fixed, and firm abdominopelvic mass with no signs of fluid collection in her peritoneal cavity. There was a continuous leakage of urine through her vagina but a leak point was unidentified. Her vaginal canal was filled with a 4 cm by 5 cm, oval and stony hard mass.

Laboratory findings, complete blood count, and organ function tests were in the normal range. An abdominal ultrasound was difficult to perform as there was only shadowing in her pelvis from the bony structures and our patient was incontinent, therefore, she was unable to retain urine.

**Fig. 1:**
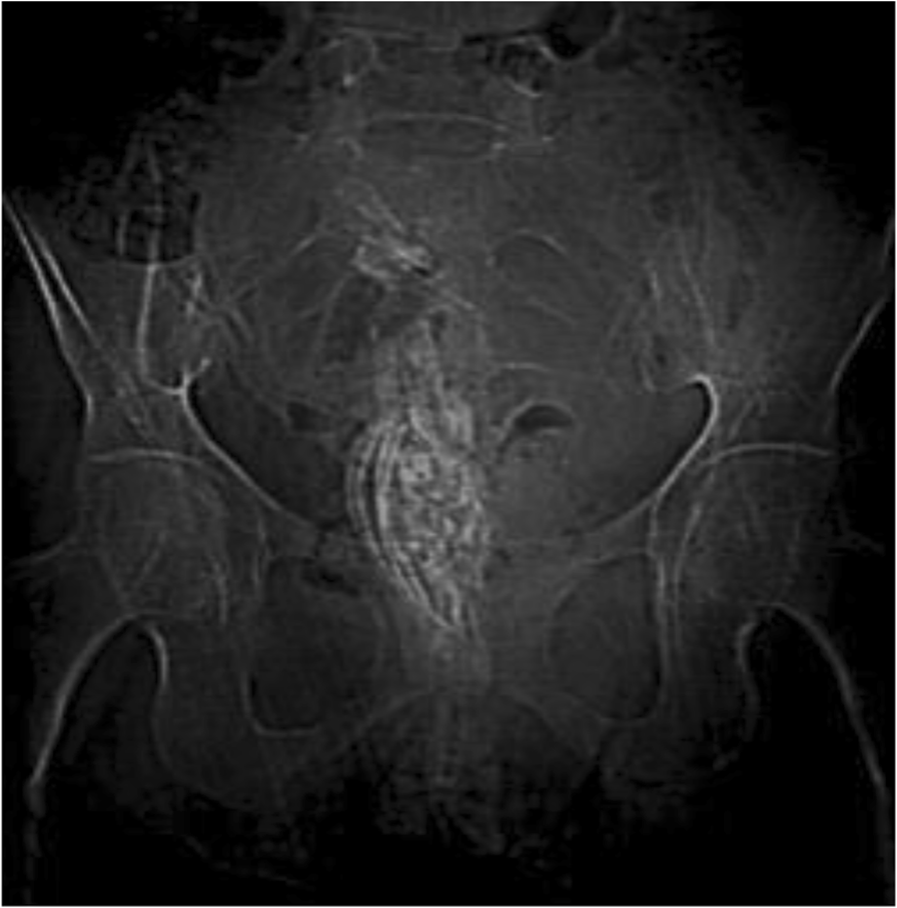
Scanogram shows calcified tubular fetal parts in the pelvis

**Fig. 2:**
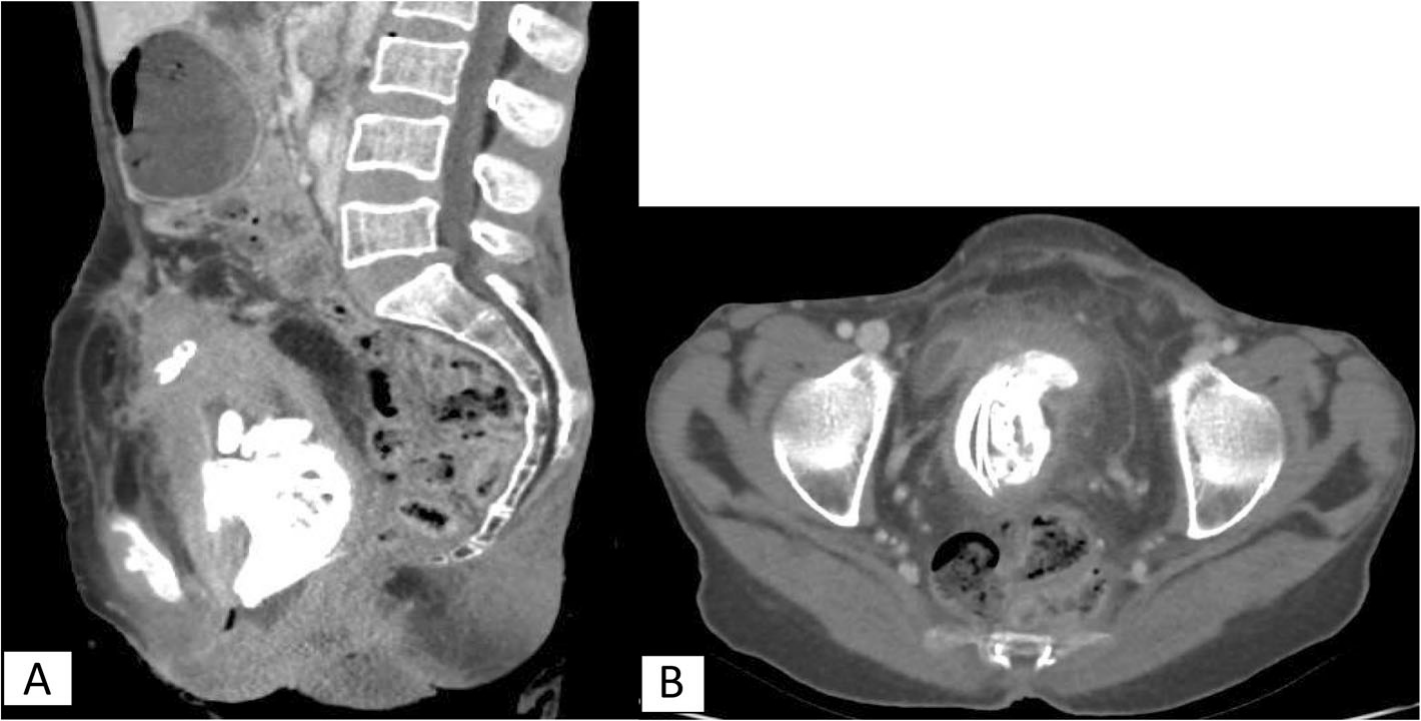
Post-contrast abdominal computed tomography images. **a** Sagittal image showing intrauterine fetal bony parts and focal adhesion of the uterus to the anterior abdominal wall. **b**. Axial image showing multiple bony structures of the fetus and displaced urinary bladder anterolateral

Abdominopelvic computed tomography (CT) findings: On the scanogram, multiple calcified tubular fetal parts were demonstrated in the pelvis of our patient (Fig. 1). On the post-contrast images, the uterus was enlarged and the fundus extended up to the level of the umbilicus. Well-formed tubular bony parts were detected within the uterine cavity which had compressed and displaced the bladder. A diffuse pelvic and lower abdomen inflammation and focal adhesion of the uterus to the abdominal wall were additionally noted (Fig. 2a, b).

In January 2019, our patient underwent exploratory laparotomy. It was difficult to enter the peritoneal cavity because of the extensive adhesion of her uterus to the anterior abdominal wall. Her uterus was completely covered by the omentum forming a complex mass. Therefore, the peritoneal cavity was entered at the level of the epigastrium. Lysis of the adhesion and omentectomy were done starting from above to free her uterus from the anterior abdominal wall and the omentum which had gone into the uterine cavity through the anterior uterine wall rupture site.

**Fig. 3:**
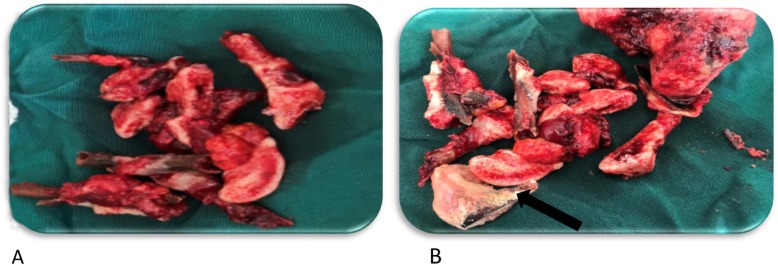
**a**, **b** Multiple fragments of lithopedion after surgical removal abdominally and vaginally (*black arrow*)

**Fig. 4:**
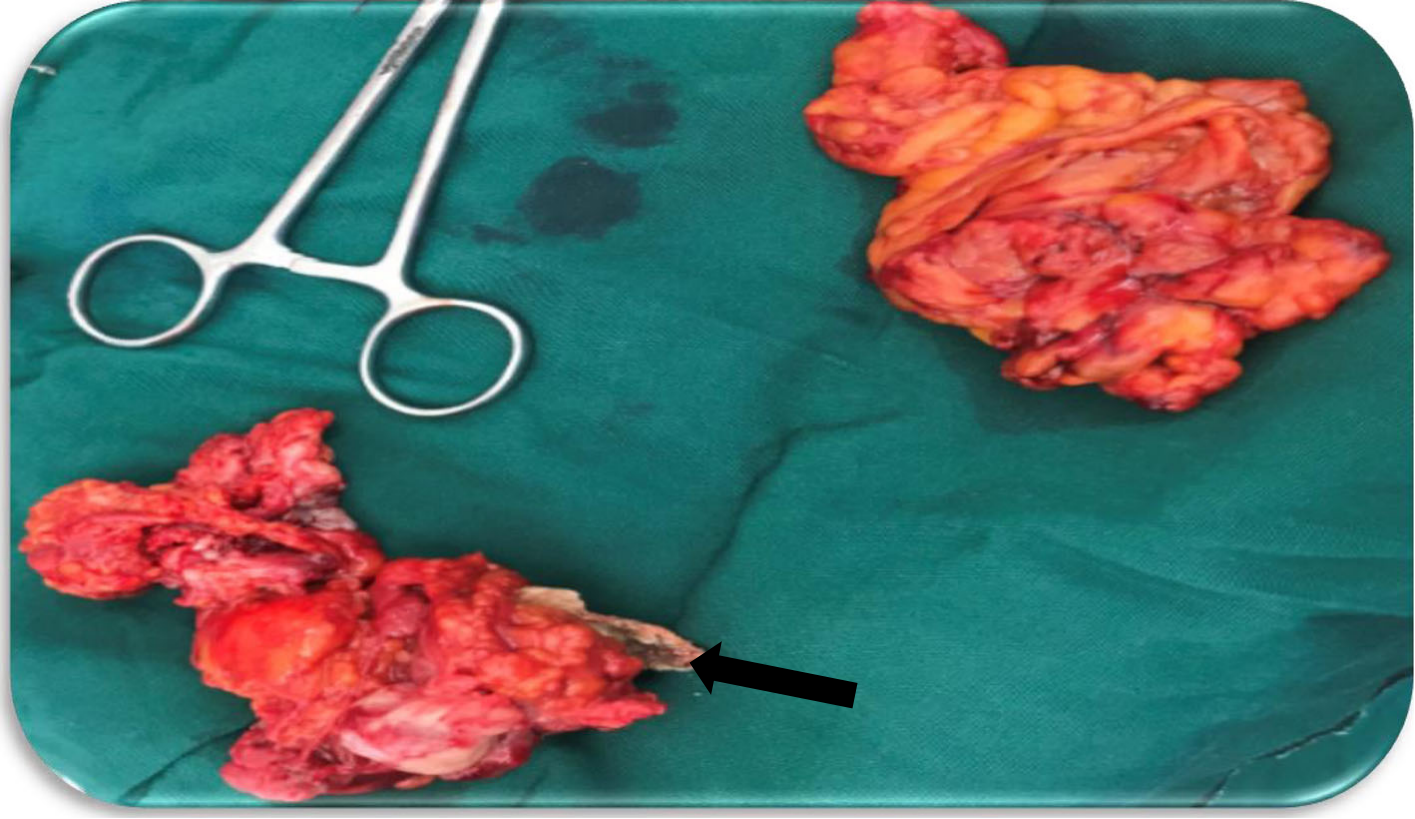
Postoperative picture of the uterus containing fetal bone (*black arrow*), adnexa, and omentum

Multiple pieces of old necrotic fetal bones (Fig. 3a, b) were extracted from her uterine cavity and those that were difficult to separate from the complex mass were removed along with the mass, the uterus, and adnexa (Figs. 3b and 4). Removal of the mass abdominally freed the fetal skull bones (Fig. 3b) in the vagina; the fetal skull bones were later easily removed transvaginally. A 3 cm defect was identified on the upper anterior vaginal mucosa and bladder wall that was obscured by the fetal skull. A Foley catheter was left *in situ* to drain urine but it could not prevent leakage through the fistula.

She had a fairly stable postoperative course and was discharged with a referral to a fistula hospital for repair of the VVF.

## Discussion

Lithopedion, or stone child, represents a condition in which a dead fetus is retained and calcified [3]. The reasons for the progression of a lithopedion include an extrauterine pregnancy that has escaped medical detection, fetal death after 3 months of pregnancy, a fetus that has remained sterile, and local conditions that favor calcium deposition [4]. When a dead fetus is too big to become reabsorbed by the mother’s body, the immune system treats it as a foreign body. This induces calcium-rich substance deposition on the fetus that will, in due course, turn the fetus body into stone [5].

This rare entity occurs with an unpredictable clinical course. Most of the cases may remain asymptomatic for a long period of time or present with persistent or recurrent abdominal pain and obstructive symptoms of the bowel and urinary system. It can also present with complications such as pelvic abscess, cephalopelvic disproportion in future pregnancies, extrusion of fetal parts through the abdominal wall, rectum or vagina fistula formation, tubal infertility, and lithopedion-induced malignancy [2, 6, 7].

This case report might not represent a classic example of a lithopedion as it is not merely an extrauterine pregnancy, but there have been five cases of lithopedion reported in the horn of a bicornuate uterus [8]. Uterine rupture due to obstructed labor is a condition that may cause the death of both the fetus and the mother unless it is surgically treated. After a uterine rupture, the fetus will be extruded into the peritoneal cavity, and fetal parts can be palpable on examination [9]. This case is unusual in that one part of the dead fetus (skull) was in the vagina and the remaining parts were located in the pelvis. A complex mass was created by the omentum that entered into the uterine cavity through the rupture site during the acute phase. This condition saved the woman’s life.

Since most literature on this subject are case reports, it is not clear what the best diagnostic tools might be or the most appropriate management approach. However, surgery has been the chosen treatment in most of the reported cases.

## Conclusion

This case report is unusual in nature because our patient carried a dead fetus in her abdomen for more than two decades and presented with VVF which occurred after a uterine rupture.
